# Exploiting Advanced Hydrogel Technologies to Address Key Challenges in Regenerative Medicine

**DOI:** 10.1002/adhm.201700939

**Published:** 2018-01-09

**Authors:** Daniel A. Foyt, Michael D. A. Norman, Tracy T. L. Yu, Eileen Gentleman

**Affiliations:** ^1^ Centre for Craniofacial and Regenerative Biology King's College London London SE1 9RT UK

**Keywords:** advanced therapies, biomaterials, bioprinting, hydrogels, regenerative medicine, tissue engineering

## Abstract

Regenerative medicine aims to tackle a panoply of challenges from repairing focal damage to articular cartilage to preventing pathological tissue remodeling after myocardial infarction. Hydrogels are water‐swollen networks formed from synthetic or naturally derived polymers and are emerging as important tools to address these challenges. Recent advances in hydrogel chemistries are enabling researchers to create hydrogels that can act as 3D ex vivo tissue models, allowing them to explore fundamental questions in cell biology by replicating tissues' dynamic and nonlinear physical properties. Enabled by cutting edge techniques such as 3D bioprinting, cell‐laden hydrogels are also being developed with highly controlled tissue‐specific architectures, vasculature, and biological functions that together can direct tissue repair. Moreover, advanced in situ forming and acellular hydrogels are increasingly finding use as delivery vehicles for bioactive compounds and in mediating host cell response. Here, advances in the design and fabrication of hydrogels for regenerative medicine are reviewed. It is also addressed how controlled chemistries are allowing for precise engineering of spatial and time‐dependent properties in hydrogels with a look to how these materials will eventually translate to clinical applications.

## 1 Introduction

Hydrogels are water‐swollen polymer networks formed by cross‐linked polymer chains and are ubiquitous in nature. The slime‐producing, eel‐like hagfish releases a mucin‐based hydrogel to choke its predators,^1^ and humans rely on a hydrogel network composed of collagen and hyaluronic acid (HA) to form the vitreous humor of the eye.^2^ However, hydrogels can also be formed from synthetic polymers, opening a myriad of synthesis strategies to create materials with widely different physical, chemical, and biological properties. Because of their hydrophilicity and chemical amenability, hydrogels have long been an exciting and promising tool in biomaterials and biomedical research. Indeed, their use in applications including as soft contact lenses for correcting vision was first proposed by Czech chemists Wichterle and Lím more than 50 years ago.^3^ However, the rise of the fields of drug delivery, cell therapies, and tissue engineering (TE) over the past decades has widened the scope of their potential applications and hydrogels are now being developed to do everything from repair articular cartilage damaged by osteoarthritis,^4^, ^5^ to regenerate heart tissue after myocardial infarction.^6^

The native extracellular matrix (ECM) can be thought of as a cross‐linked, hydrophilic polymer network. Therefore, at a fundamental level, hydrogels, whether synthetic or naturally derived, are in many ways akin to the native ECM. Many hydrogels can also be cross‐linked under mild conditions, allowing for the encapsulation of live cells. Because of these features, hydrogels have been proposed as 3D ex vivo tissue models. The 3D network of hydrogels, which enable encapsulated cells to interact with their environment in all directions, often better replicates the environment cells experience within tissues compared to 2D cultures, which can force cells to adopt unnatural polarities. Hydrogels' chemical amenability also allows them to be formed with widely different physical properties, including stiffness, and biological functionalizations mediated by the incorporation of adhesive and degradable peptide sequences, which can mimic many biological and physical properties of the native ECM. Hydrogels are also being explored as therapeutic delivery vehicles. Acellular hydrogels can be designed for site‐specific slow release of drugs or other bioactive molecules, such as growth factors. And hydrogels with encapsulated cells are being developed for TE and other regenerative strategies. By modulating their physical and biological properties, hydrogels can coax encapsulated cells to form new tissues. They can also retain therapeutic cells at specific tissue sites, allowing them to mediate repair either indirectly via paracrine signaling, or directly, by differentiating and producing tissue.

Despite these exciting developments, hydrogels have also been subject to criticism. Although their hydrophilic properties are akin to that of many native tissue ECMs, early generations of hydrogels used for many biomedical applications lacked important properties of native tissues that are known to be key in directing cell behavior. Native tissues, for example, are heterogeneous in structure, respond dynamically to their surrounding environment,^7^ can often self‐heal in response to injury,^8^ and their mechanical properties tend to be nonlinear and often viscoelastic.^9^ Conversely, many standard hydrogels, particularly those formed from synthetic polymers, are static, only sparingly adaptable to cell‐mediated changes, and their mechanical properties are often linearly elastic. Moreover, many hydrogels are structurally homogeneous and cannot mimic the architectural and mechanical complexity of native tissues at multiple length scales. In general, many hydrogels also have relatively weak mechanical properties for TE applications. For example, hydrogels have been widely proposed for cartilage TE, however, their tensile and compressive properties often do not match those of the native tissue.^10^

The last decade, however, has witnessed an explosion of new chemistries, designs, and fabrication methods that have returned hydrogels to the forefront of cutting‐edge biomaterials research (**Table** **1**). Researchers are exploring a new generation of hydrogel‐based biomaterials that better act as tissue models by mimicking the time‐dependent and nonlinear properties that govern the behavior of native tissues. They are also designing materials for regeneration that interact with cells as never before. Not just delivering cells locally, but doing so in a controlled way, or designing chemistries that recruit cells to the material. 3D printing methods have also been developed to precisely control tissue architecture and cell localization within tissue‐like constructs, and for the first time, have allowed for the creation of complex tissue‐like structures with vasculature. These next generation materials require delivery methods to match. Therefore, researchers have also been exploring exciting means for in situ cross‐linking and injectable delivery. And indeed, hydrogels that aim to repair defects in articular cartilage and restore damaged heart tissue after myocardial infarction are now in preclinical and clinical trials.

**Table 1:** Table highlighting important design criteria for biological hydrogels

Design criteria	Design variables	Factors to consider
**Material**	**Type of material** • Natural: collagen, fibrin, and alginate • Synthetic: polyacrylamide, polyethylene glycol (PEG) • Hybrid materials: hyaluronic acid (HA), polypeptides	• Biocompatible • Template for tissue growth in 3D • Allow for vascularization (microporous) • Appropriate mechanical properties • Biodegradable—reabsorbs at same rate as regeneration
**Cross‐linking strategies**	**Physical (noncovalent) cross‐linking**: • Ionic interactions—charge interactions • Peptide based self‐assembly—Supramolecular structures, e.g., β‐sheets **Chemical (covalent) cross‐linking**: • Chain growth polymerization—via redox or photoinitiation (UV) • Click chemistry— e.g., Michael‐type addition • Enzyme‐mediated cross‐linking—e.g., transglutaminase and tyrosinase	• Speed of cross‐linking • Complexity of reagents • Appropriate for cell encapsulation—cytocompatible • Precise control of microstructure (supramolecular) • Cross‐linking density (network porosity) • Control of mechanical properties such as: o Elasticity/viscoelasticity o Viscosity
**Delivery methods**	• Injectable—in situ forming hydrogels • Hydrogel patch—Such as transdermal or epicardial • Implant—preformed hydrogel scaffold—e.g., bioprinted	• Invasiveness of the procedure • Target organ • Aim of treatment—drug delivery, tissue regeneration, etc.
**Biological agents**	**Cell encapsulation**: • Mesenchymal stem cells (MSC) or induced pluripotent stem cells (iPSC) • Autologous or allogenic	• Immunogenicity • Safety (following genetic manipulation) • Cost and availability
	**Biological molecules**: • Growth factors, immunomodulatory, gene therapy	• Dosage • Choice of biological agent(s) • Timing (sustained release vs temporally specific)

**Figure 1:**
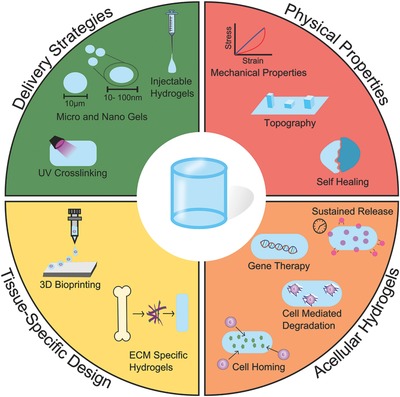
Summary of state‐of‐the‐art strategies for hydrogel design and fabrication and their applications in regenerative medicine. Advancements in both biology and material science have allowed for the development of complex regenerative strategies. Green: Researchers are designing hydrogels with various delivery strategies tailored for each biological application. Red: Our increased understanding of mechanobiology is driving the development of hydrogels that can aid biologists in understanding these fundamental processes, and allow researchers to exploit them to drive cell response for regeneration. Yellow: Advanced manufacturing technologies are allowing for the development of hydrogels with tissue‐specific architectures and biological functionalities. Orange: Acellular hydrogels are being developed to both deliver relevant biological molecules and direct host tissue response.

Here, we review some of the state‐of‐the‐art advances in the design and fabrication of hydrogels for regenerative medicine (**Figure** **1**). We specifically focus on where exciting new chemistries and manufacturing techniques are allowing researchers to make materials that better mimic the native tissue. We also address how controlled chemistries are allowing for more precise engineering of spatial and time‐dependent properties in these hydrogels with a look to how these materials will eventually translate to clinical applications.

## 2 Hydrogels as Ex Vivo Tissue Models

It was not long after George Otto Gey managed to culture Henrietta Lacks's cervical cancer cells in a dish^11^ that researchers realized that cells behave differently in the body than they do on tissue culture plastic.^12^ Many cells on 2D surfaces adopt unnatural polarities and create large focal adhesion plaques, behaviors (among a myriad others), which fundamentally differ when cells are within native tissues.^12^ Therefore, to fundamentally understand how cells respond in health and disease to a variety of stimuli, it is important to develop culture systems which better mimic cells' normal 3D environments. The 3D structure and ECM‐like properties of hydrogels make them one of the best tools biologists have for doing exactly this.

Over the past 15 years, the fields of cell and stem cell biology have uncovered an increasing role for physical properties of the ECM in directing cell behaviors. Indeed, materials that control cell morphology, elastic, and viscoelastic properties of cells' substrates, and micro‐ and nanoscale topographies, among other factors, have been shown to play important roles in directing stem cell differentiation and driving other fundamental cell behaviors. As our understanding of how physical properties of the ECM direct stem cell fate and tissue formation have grown, we have witnessed a concomitant development of biomaterials that mimic such properties. However, translating behaviors on 2D surfaces to 3D tissue‐like platforms have revealed additional complexities. In 3D, not only do substrate stiffness and topography play important roles, but also cell‐mediated matrix degradability, cell migration, and physical constraint.^13^, ^14^ The field is currently developing new hydrogels that allow us to understand the interplay between these factors, and how they independently and synergistically direct cell behavior.

### 2.1 Incorporating Adhesive Motifs

With the notable exception of blood cells, most cells in the body are anchorage dependent. That is, they must adhere to a substrate to survive. Anchorage‐dependent cells deprived of a substrate on which to attach will undergo a specialized form of apoptosis called anoikis.^15^ Engaging integrin receptors—cells' transmembrane structures that mediate attachment to extracellular substrates—will not rescue viability^16^ because most cells have to be physically tethered to a surface to survive. In vitro, this substrate is normally tissue culture plastic, a plasma‐treated polystyrene surface that adsorbs proteins onto which cells attach.^17^ In the body, this substrate is often the ECM, a network of insoluble protein biopolymers, which can contain binding sites that mediate interactions with cells. Hydrogels formed from many ECM‐derived biopolymers such as collagen contain abundant binding sites that mediate interactions between the hydrogel and encapsulated cells. However, synthetic polymers such as poly(ethylene glycol) (PEG) and even biologically derived matrices such as alginate do not. Therefore, adhesive motifs need to be incorporated into hydrogels to allow attachment to their surroundings and mediate cell viability.

Hydrogels intended for cell encapsulation that are lacking in their own binding motifs are often chemically modified to display them. Standard strategies include tethering proteins or peptides with integrin‐binding sequences to the polymer backbone of the hydrogel.^18^, ^19^ For example, multiarm PEG molecules are often conjugated to enable some arms to engage in cross‐linking reactions while others present pendant adhesive motifs. Typical natural ECM components incorporated into hydrogels include collagen, fibronectin, and laminin. While common adhesive peptide sequences include fibronectin‐derived RGD and LDV and laminin‐derived IKVAV and YIGSR.^20^ Cell–ECM binding interactions can trigger specific signaling cascades within the cell, including those that control differentiation. This behavior is mediated through integrins, which only recognize and interact with specific peptide sequences within the ECM. For example, although human marrow stromal cell (often referred to as mesenchymal stem cells, MSC) express a wide range of integrins, including α1, α2, α3, α4, α5, αV, β2, and β1, it is their engagement with RGD sequence‐containing peptides that supports their long‐term viability. However, as human MSC (hMSC) differentiate, their integrin expression patterns change, and the binding of specific motifs drives lineage specification. For example, the IKVAV binding peptide sequence, which engages α4β1 and α6β1 integrins, promotes osteogenesis to a greater extent than that induced by YIGSR and RRETAWA binding sequences. Similarly, both the IKVAV and RRETAWA sequences, which bind to α4β1/α6β1 and α5β1 integrins, respectively, are more conducive for adipogenesis than the RGD binding sequence alone.^19^, ^21^ Although beyond the scope of this review, the reader is referred to excellent summaries of the role of integrin binding in controlling cell behavior.^22^

Moreover, while the composition of the ECM and ECM‐mimicking peptides incorporated within hydrogels is clearly important in modulating cell attachment and downstream cell signaling, the spacing of integrin binding motifs may also be important. On 2D surfaces, cells are known to be highly sensitive to interligand spacing. For example, increasing interligand distances from 58 to 73 nm has been shown to alter cell morphology.^23^ Moreover, smaller interligand spacings appear to induce a bias in MSC differentiation toward osteogenesis.^24^ Most 3D hydrogels allow for modulation of overall ligand density, and there is evidence that PEG hydrogels modified with dangling RGD‐binding sequences do not support cellular interactions below certain concentrations;^25^ however, the role of precise ligand spacing, akin to those that have been examined on 2D surfaces, has only recently been explored in 3D. Pashuck et al. synthesized peptides that utilized a β‐sheet motif to self‐assemble into nanofiber hydrogels.^26^ As the β‐sheet peptide's backbone spacing was known, adhesive epitopes (RGDS and PHSRN) were controllably spaced at distances between 0.7 and 6.2 nm. Endothelial cells encapsulated within hydrogels with spacings of 3.2 nm, which mimicked the spacing found in fibronectin, showed significant upregulation of the α_5_ integrin subunit and adopted more spread morphologies when compared to cells encapsulated within hydrogels with 6.2 nm spacing. Ideal networks with orthogonal geometries are similarly being explored to further unravel the role, if any, of 3D spacing cues in directing other cell responses, such as differentiation.^27^

### 2.2 Controlling Cell Morphology

2D cell shape has long been known to regulate cell behaviors. Watt et al.^28^ determined that cell morphology regulated epidermal cell lineage commitment in the 1980s, and McBeath et al.^29^ reported that hMSC morphology regulated a fate switch between adipogenesis and osteogenesis through Ras homolog gene family member A/Rho‐associated, coiled‐coil containing protein kinase 1 (RhoA/ROCK) signaling. Many of these findings were made possible by a technique called microcontact printing, in which 2D patterns of adhesive motifs are stamped onto an otherwise nonadhesive surface, allowing precise control of cell size and shape. Using this technique, they and others established that in MSC, spread cell morphologies promote cytoskeletal tension, which upregulates osteogenesis, and round morphologies promote adipogenesis.

Mechanoregulation of MSC fate has recently been associated with the downstream hippo pathway effectors Yes‐associated protein (Yap) and transcriptional co‐activator with PDZ‐binding motif (Taz).^30^ Spread MSC morphologies promote nuclear localization of Yap/Taz, while restricting cell spreading, inhibits cytoskeletal tension, excludes Yap/Taz from cell nuclei, and prompts MSC to adopt adipogenic phenotypes. However, while Yap/Taz signaling appears to be a key mediator of the conversion of physical to chemical signals, other candidates such as lamin‐A and the retinoic acid pathway are also thought to be important in the mechanoregulation of a plethora of cell responses.^31^ Stem cells other than MSC have similarly been shown to be mechanoresponsive. Changes in actin cytoskeleton organization, for example, have been shown to induce epidermal stem cell differentiation.^32^ Indeed, when spreading is restricted, keratinocytes differentiate by regulating serum response factor (SRF). SRF then targets FOS and JUNB, members of the AP‐1 family of transcription factors, which are known to play important roles in epidermal terminal differentiation.^32^ Although a more detailed discussion is outside the scope of this review, putative signaling mechanisms by which cells sense and respond to their physical environment have been previously explored.^33^

In addition to showing that cell size influences differentiation, researchers have also used microcontact printing to demonstrate that pattern shape, and specifically shape perimeter, also directs lineage specification. MSC on more rounded patterns that have smaller perimeters adopt adipogenic phenotypes, while MSC on patterns with steeper angles that have larger perimeters, such as star shapes, promote osteogenesis, even when total cell area is kept constant.^34^ For a comprehensive review, the reader is referred to an excellent review by Vogel and Sheetz.^35^

While control of cell morphology in 2D is relatively straightforward and has revealed clear effects on MSC lineage specification, controlling cell morphology in 3D hydrogels is more complex. On 2D surfaces, cells face no external barriers when assuming their morphologies. However, in both native tissues and within 3D culture, cell morphology is limited by the presence of the ECM or encapsulating material. In 3D hydrogels, morphology is generally controlled through degradation either by incorporating enzyme‐mediated degradation into the hydrogel, or directly 3D patterning degradative motifs. To change their morphology in this context, cells often degrade their surroundings by secreting enzymes, which target specific proteins in their ECM. Within hydrogels formed from naturally derived polymers such as gelatine, collagen, and fibrin, as well as polysaccharides such as HA,^36^ cells can often degrade their surroundings as they do in native tissues. However, within synthetic 3D hydrogels, control of cell shape is often achieved by cross‐linking the network with peptides containing sequences that can be cleaved by cell‐secreted enzymes. In nondegradable hydrogels, cells will often adopt round morphologies, even when presented with adhesive motifs. However, the incorporation of matrix metalloproteinase (MMP)‐degradable peptide sequences allows cells to adopt spread morphologies.^37^ Controlling cell morphology through degradation can have a profound impact on differentiation. Indeed, hMSC encapsulated within RGD‐modified HA‐based hydrogels that are degradable adopt spread morphologies, generate cytoskeletal tension and undergo osteogenic differentiation,^38^ while limiting degradation prompts cells to adopt adipogenic phenotypes.

**Figure 2:**
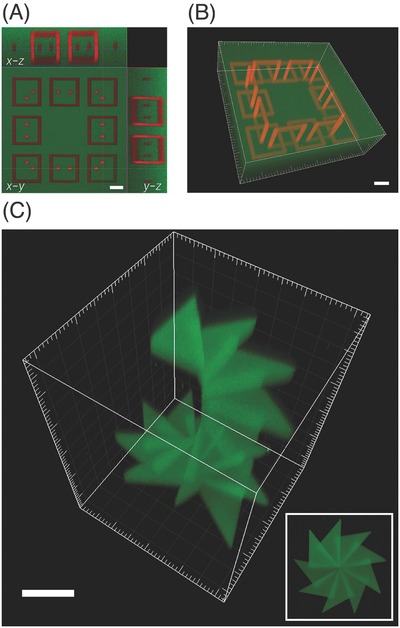
Microscale photoreversible patterning of proteins within 3D hydrogels. A,B) Fluorescence confocal microscopy images of dual‐protein patterning within hydrogels. Hydrogels were patterned with covalently immobilized interlocking chains of red protein while surrounding areas were labelled with a green protein. Scale bars = 50 µm. C) Hydrogel with 3D patterned protein in a staircase pattern. Patterning was achieved in 3D using focused laser pulses and by varying the multiphoton laser‐scanning conditions, resulting in highly ordered positioning of proteins. Scale bar = 150 µm. Adapted with permission.^[186]^ Copyright 2015, Nature Publishing Group.

The alternative strategy to control cell morphologies and migration within 3D hydrogels is to spatially control the availability of degradable and adhesive sites. Recently, this has been achieved with the use of light‐sensitive chemistries in combination with patterned UV irradiation and laser exposure (**Figure** **2**).^39^ Researchers accomplished this by incorporating nitrobenzyl ether derivatives into hydrogels, which cleave upon exposure to 365 nm light. The light‐sensitive groups are then linked to adhesion and/or cross‐linking components of the hydrogel. By carefully focusing a laser at specific locations within the 3D hydrogel, degradation can be induced by breaking the cross‐links that hold the hydrogel together, or cell adhesion can be inhibited by cleaving cell‐adhesive peptides from the hydrogel network. Encapsulated cells detect the light‐mediated degradation by altering their migratory behavior; and sense changes in adhesive ligands by modifying their morphologies. This technology is particularly promising because it allows for patterning of cell adhesion and degradability in a 3D spatiotemporal manner with micrometer‐scale resolution.

### 2.3 Modulating Substrate Stiffness

In 2006, Engler et al.^40^ reported that 2D substrate stiffness had a profound effect on the lineage commitment of hMSC in the absence of chemical induction. Soft substrates that mimicked the stiffness of brain tissue promoted neurogenesis, stiffer substrates more akin to skeletal muscle prompted cells to become myogenic, and stiff substrates that matched the mechanical properties of the developing osteon drove cells to adopt osteogenic phenotypes. The implication was that cells physically “felt” the stiffness of their underlying matrix and used this information as an important driver of lineage specification. Although only shown on 2D substrates, this finding opened the possibility that stiffness itself could be exploited to direct stem cell differentiation. As a result, there has been tremendous interest in developing hydrogel systems that allow researchers to understand the role of 3D stiffness in directing the differentiation of MSC in ex vivo tissue models.

Although the role of 2D stiffness in regulating stem cell fate has been clear for more than a decade,^40^, ^41^, ^42^ the role of stiffness in directing MSC lineage specification in 3D is more complex. Using a nondegradable, RGD‐modified, ionically cross‐linked alginate‐based hydrogel, Huebsch et al. showed that like on 2D surfaces, MSC responded to the stiffness of their surrounds in 3D.^43^ That is, stiffer hydrogels promoted osteogenesis and softer adipogenesis. However, unlike in 2D, stiffness did not impact cell morphology, and all cells maintained round morphologies independent of hydrogel stiffness. They instead showed that differentiation depended on the clustering of integrin‐binding motifs within the hydrogels, which was dependent on substrate stiffness.

However, in degradable hydrogels formed from RGD‐modified, methacrylated HA, degradation‐mediated cellular traction was found to direct MSC differentiation, independent of substrate stiffness.^38^ That is, hMSC adopted adipogenic phenotypes over a wide range of stiffnesses (4.91–91.64 kPa) in nondegradable RGD‐modified methacrylated HA hydrogels, but when hydrogels were modified to allow cell‐mediated degradation, hMSC generated cytoskeletal tension (hydrogel stiffness ≈3–5 kPa) and differentiated down the osteogenic lineage. The authors attributed these findings to the relative differences between ionically and covalently cross‐linked hydrogels. However, the role of stiffness in the differentiation of MSC in 3D hydrogel cultures remains controversial and other factors could indeed also be playing important roles. For instance, time‐dependent effects in hydrogel systems could confound results. Cells modify their surrounding matrix by secreting proteins extracellularly over time when encapsulated in 3D.^44^ Moreover, 3D hydrogel matrices that allow for cell‐mediated degradability will undergo time‐dependent changes in local and perhaps bulk stiffness as the hydrogel degrades, which may influence cell response.

In summary, utilizing modifiable hydrogels to understand the contribution of stiffness to lineage specification can be fraught. While nondegradable systems will likely provide insight into the differentiation of cells that reside in tissues in which matrix turnover is slow, degradable systems may better mimic native tissue niches that experience quicker ECM turnover. Matrix degradation is known to play central roles in development, stem cell differentiation, and tissue formation. For example, in the developing embryo, cells migrate through 3D matrices, undergo cell shape changes concomitant with differentiation, and remodel their ECM. These behaviors are, in many cases, dependent on the degradability of the native ECM.^45^ When ECM degradation in the developing mouse embryo is repressed by MMP inhibitors, morphogenesis and development of oil‐induced deciduomas are slowed, and changes take place in precursor stromal cell differentiation and expansion.^46^ Cell‐mediated matrix degradation is also important in wound healing. When matrix degradation was inhibited by the broad‐spectrum MMP inhibitor BB‐94 in full‐thickness skin excisional wounds in rats, myofibroblast formation, stromal cell proliferation, blood vessel formation, and epithelial wound coverage, key components of healthy skin wound healing, were all delayed.^47^ In short, many tissues are highly dynamic and degradation and ECM remodeling are key tools they utilize to maintain homeostasis. Therefore, incorporation of this feature into ex vivo models may be important to fully understand and exploit tissue regeneration.

### 2.4 Integrating Time‐Dependent and Self‐Healing Properties

Many tissues in the body, particularly soft tissues, exhibit viscoelastic mechanical properties. That is, they behave simultaneously as both an elastic material and a viscous fluid. Viscoelastic materials will continue to deform, or creep, if left under an applied load, or undergo stress relaxation, whereby they exert less stress over time, when placed under a constant deformation.^42^ Such properties are well described in musculoskeletal tissues such as ligament and tendon where these behaviors play important roles in normal joint function.^48^

Until recently, most synthetic hydrogels examined for directing cell behavior in response to mechanical properties such as stiffness were linearly elastic. That is, they possessed a linear relationship between stress and strain, and they returned to their original shape upon unloading, without loss of energy. However, it is now becoming apparent that cells, both on 2D surfaces and when encapsulated within 3D hydrogels, respond not only to the elastic but also viscoelastic properties of their underlying or surrounding matrix.^49^, ^50^ Chaudhuri et al. recently showed that a human osteosarcoma cell line (U2OS) responded differently when cultured on the surface of viscoelastic ionically cross‐linking alginate hydrogels compared to when they were on purely elastic hydrogels of the same initial stiffness that were formed from covalently cross‐linking the alginate.^49^ Indeed, counter to the intuitive expectation that cells would integrate the modulus of a relaxing substrate over time and behave as if they were on a softer substrate, U2OS cells actually spread more on substrates that underwent stress relaxation. The authors suggested that cells' ability to respond to viscoelasticity is likely a fundamental biological property.

Similarly intriguing observations were reported when cells were encapsulated within 3D viscoelastic hydrogels.^51^ In RGD‐modified alginate hydrogels, the proliferation and morphology of encapsulated 3T3 fibroblasts were highly dependent on the time scale of stress relaxation. That is, in matrices that underwent fast relaxation, cells tended to spread more and proliferate, while slowly relaxing materials prompted cells to adopt round morphologies and inhibited proliferation. Similarly, when murine MSC were encapsulated within fast‐relaxing hydrogels, they differentiated to osteoblasts that formed a mineralized collagen type I‐rich matrix, but in slowly relaxing hydrogels, MSC adopted adipogenic phenotypes. The authors attributed these observations to the ability of encapsulated cells to cluster RGD ligands in the faster‐relaxing hydrogels. They also argued that in fast‐relaxing hydrogels, MSC were more able to mechanically remodel their surrounding matrix. Interestingly, the authors correlated increased Yap nuclear localization with faster‐relaxing hydrogels, independent of hydrogel stiffness. However, unlike in 2D systems where nuclear localization has been correlated with osteogenesis,^30^ substrate viscoelasticity‐mediated nuclear translocation of Yap, did not itself direct lineage specification.

In addition to viscoelastic hydrogels, much interest has also been focused on creating viscoelastic hydrogels that are also self‐healing, and so can mimic repair processes that take place in native tissues. These hydrogels are typically composed of macromolecules that are noncovalently bonded together via molecular recognition motifs such as hydrophobic interactions, π–π interactions, hydrogen bonding, metal chelation, or van der Waals interactions.^52^ Various mechanisms have been exploited to create self‐healing hydrogels, but the basic premise is that interactions between individual molecules are locally dynamic, but the bulk hydrogel is globally stable. The noncovalent and dynamic nature of these interactions not only make these hydrogels self‐healing, but also easily injectable and shear thinning. Shear thinning materials can protect encapsulated cells against fluid shear forces generated during injection by “un‐cross‐linking” under shear and re‐establishing cross‐links when shear is removed.^53^ A key advantage of shear thinning hydrogels formed through physical interactions is that they can be formed ex vivo, prior to injection. In this context, the influence of the surrounding tissues on hydrogel gelation is negligible, in contrast to hydrogel systems with liquid precursors, whose gelation may be affected by various constituents of the native milieu. Self‐healing hydrogels are yet to be fully exploited to understand cell‐material interactions relevant to TE and regenerative medicine. For a comprehensive review of self‐healing biomaterials, the reader is referred to an excellent review by Webber et al.^52^

## 3 Hydrogels with Tissue‐Specific Mimicry and Functionality

**Figure 3:**
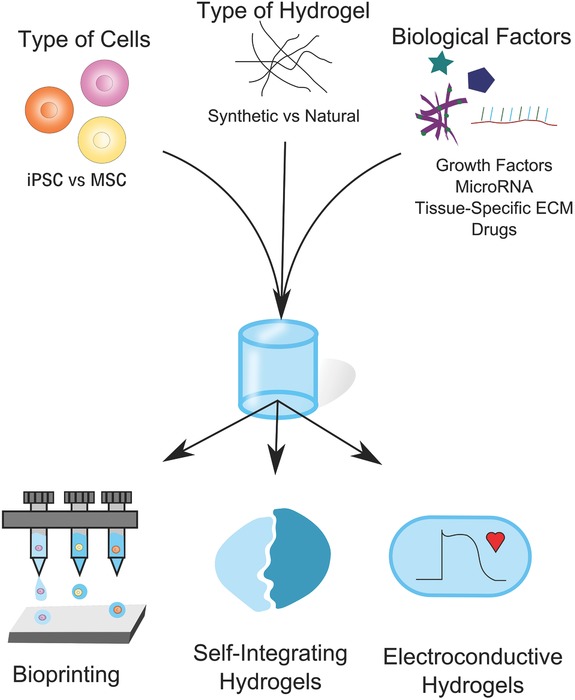
Tissue‐specific hydrogel design considerations. When developing tissue‐specific hydrogels, a number of factors should be considered. For example, the choice of cell type and biological factors will be dictated by the biological application. However, the emergence of new technologies, such as those that allow for the creation of patient‐specific stem cells, for example iPSC, may allow for additional opportunities. Hydrogel material choice is not always straightforward and may be dictated by a range of factors, such as amenability to bioprinting, the need to form a tissue interface or the necessity of tissue‐specific functionality.

In addition to developing materials that can be used as ex vivo tissue models, there is also tremendous interest in using hydrogels directly in TE. TE aims to treat a myriad of diseases by replacing lost/damaged tissue with living constructs created in the laboratory. The applications of TE range from restoring tissue lost to myocardial infarction, to filling bone defects with cell‐laden scaffolds that respond to load and remodel over time. However, the repair/regeneration of complex tissues and organs requires approaches that are specific to each tissue. When designing a hydrogel that can mediate tissue repair, several parameters are key to consider, including the tissue architecture, mechanical and biological cues, and cell type (**Figure** **3**). Indeed, advances in all three areas are likely key in ensuring effective regeneration.

### 3.1 Incorporating Tissue‐Specific Architectures into Hydrogel Design

Whereas many early generations of hydrogels were homogeneous, isotropic structures that bore little resemblance to complex native tissues, the design of tissue‐specific architects for TE has taken a leap over the past decade with the advent and growth of 3D bioprinting. Bioprinting is a computer‐assisted technology, which can assemble tissues‐like constructs through precise spatial localization of biological materials in 3D. Bioprinting technologies utilize “bioinks” often composed of different hydrogels, with or without encapsulated cells.^54^

**Figure 4:**
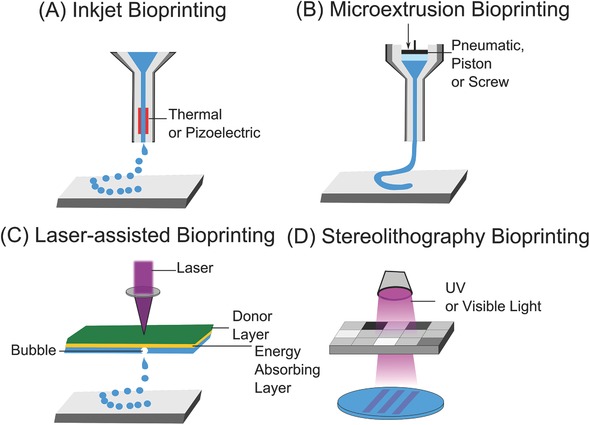
Examples of different bioprinting methods. A) Inkjet bioprinters deposit small droplets of hydrogel and cells to build tissue layer‐by‐layer. B) Microextrusion bioprinters deposit a cell‐laden liquid solution via pneumatic or manual force. C) Laser‐assisted bioprinting uses a laser to rapidly heat a donor layer (green), which forms a bubble propelling the bioink onto the substrate. D) Stereolithography bioprinters use UV or visible light to selectively cross‐link bioinks layer by layer to build a 3D construct.

Within the field of bioprinting several different methods are available, many of which are amenable to hydrogel technologies. The most common include microextrusion, inkjet, and light‐induced methods, which include stereolithography and laser‐assisted bioprinting (LAB) (**Figure** **4**). For a detailed overview, the reader is referred to excellent reviews in this area.^54^, ^55^, ^56^ Of these technologies, inkjet bioprinting was the first to be developed.^55^ Advantages of this system include its high printing speed and low cost; however, thermal and mechanical stresses can damage encapsulated cells.^54^, ^57^ Another limitation of inkjet systems is their relatively poor capacity to handle materials of high viscosities, which can limit their ability to print hydrogels with high cell densities.^54^, ^55^ Inkjet printing has been shown to be effective in the regeneration of both skin^58^ and cartilage.^59^, ^60^ For example, Markstedt et al. used a nanocellulose bioink to produce precise anatomically shaped cartilage structures such as the human ear and sheep meniscus.^60^

Microextrusion printing works on a similar basis to inkjet, however, it allows materials of higher viscosities to be deposited. This is important as it allows for the deposition of high‐density cell solutions, which are key in replicating many highly cellular native tissues.^54^ This strategy has been used to print aortic valve conduits,^61^ vascular grafts,^62^ and cartilage constructs,^63^ among other tissues. LAB systems are lauded for their high resolution as they can produce resolutions on the microscale (≈10 µm), which is important for mimicking many structures of native tissues at or near the scale of the single cell.^64^ An advantage is that these systems are nozzle free, which precludes issues with clogging that have been problematic with the other bioprinting technologies.^54^ Stereolithography, like LAB, uses light to print and works by selectively solidifying bioinks in a layer‐by‐layer process.^55^ While lacking the high resolution of LAB methodologies (≈25 µm), this layer‐by‐layer process can often increase printing speeds.^56^

In situ printing, in which materials combined with cells are printed directly into a tissue defect (as opposed to requiring later surgical implantation) is also emerging as a method to create materials in situ with precise architectures to drive tissue‐specific repair. In comparison to classic bioprinting strategies, in situ bioprinting has several advantages, most notably of which is that it potentially allows for the fast, direct delivery of cells. This style of printing is often envisioned for future clinical applications, in which fully automated robotic printers controlled by surgeons directly print architecturally controlled, cell‐laden constructs at the site of injury. In situ printing of amniotic fluid‐derived stem cells and MSC has proven effective in mediating wound closure in a severe skin wound model.^58^ Work by Keriquel et al. have similarly demonstrated a LAB‐based bioprinting system in which in situ printing of MSC collagen/hydroxyapatite bioink was able to stimulate bone regeneration in a calvarial defect model in a mouse.^65^ The authors were able to print precise scaffold patterns into the site of injury, and cells within the printed scaffold maintained good cell viability.^65^

**Figure 5:**
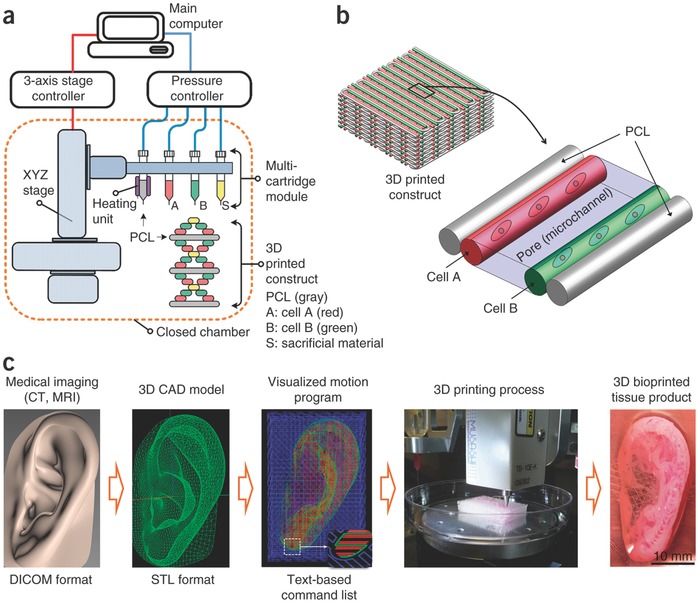
Human‐scale bioprinting. To print constructs of sufficient size for eventual translation into humans, Kang et al. developed an integrated tissue‐organ printer (ITOP) system in which large, tissue‐specific constructs could be printed. a) The ITOP system comprised three major units: i) a three‐axis stage controller, ii) a dispensing module composed of multiple cartridges and a pneumatic pressure controller, and iii) a closed acrylic chamber with a humidifier and temperature regulator. b) Using this bioprinter, 3D scaffold architectures could be printed with both multiple cell types and a PCL polymer to ensure structural rigidity. c) 3D CAD models were generated from medical image data; and using CAD/CAM processing, the printer could be used to create complex 3D structures, including a 3D human‐sized ear. Reproduced with permission.^66^ Copyright 2016, Nature Publishing Group.

While bioprinting allows for specific design of tissue constructs, one drawback of the technology has been its scalability. That is, there has been a limit thus far in printing large, structurally sound, biological constructs that are needed to repair/replace many tissues.^66^ However, one pioneering study demonstrated an integrated tissue‐organ printer in which human‐scale tissue constructs could be printed (**Figure** **5**).^66^ This bioprinting system simultaneously dispensed composite cell‐laden hydrogels consisting of fibrinogen, HA, gelatin and glycerol, alongside a synthetic biodegradable polymer and an outer sacrificial acellular hydrogel mold. This mold provided the tissue constructs with enough rigidity to ensure the construct retained its shape, but could then be easily removed. A lattice of microchannels was also incorporated into the design to allow for nutrient and oxygen diffusion within the printed construct.^66^ The authors also implemented CT and MRI imaging to help design the scaffold. Raw imaging data was processed using computer‐aided manufacturing tools and mathematical modeling to produce 3D rendered models.^66^ This allowed them to capture architectural intricacies, enabling them to print calvarial bone, ear, cartilage, and skeletal muscle.

**Figure 6:**
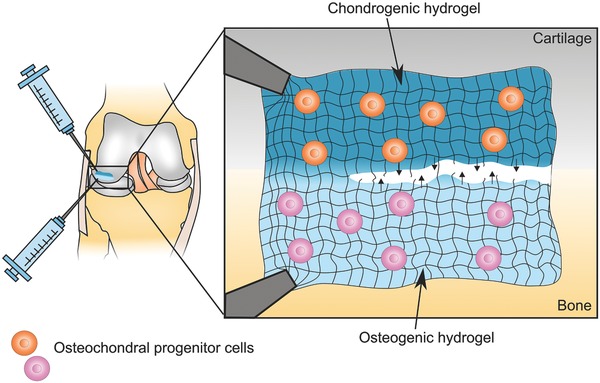
Injectable self‐integrating hydrogels for osteochondral repair. Schematic illustration of a self‐healing hydrogel that could potentially be used to repair an osteochondral defect. Two tissue‐specific hydrogel formulations made from the same bulk material are injected into the damaged cartilage–bone interface. The shear‐thinning properties of the hydrogels would allow for easy injection of the cell‐containing solutions. Their self‐healing chemistries would then foster the formation of a seamless transition between the chondrogenic and osteogenic hydrogel formulations. The chondrogenic hydrogel could, for example, contain osteochondral progenitor cells or chondrocytes and chondrogenic factors such as transforming growth factor β (TGFβ ), BMP and/or insulin‐like growth factor (IGF) to promote cartilage‐like ECM production. Whereas the osteogenic hydrogel might include nanoscale hydroxyapatite particles and VEGF to enhance bone regeneration.

Beyond bioprinting, other methods have also shown promise in integrating tissue‐specific architecture into hydrogels. One particularly promising area is that of self‐healing/self‐integrating hydrogels. These hydrogels are based on dynamic chemistries and thus are potentially exciting for a variety of TE strategies. Hsieh et al. investigated self‐healing hydrogels to induce blood capillary formation. An injectable composite hydrogel was synthesized from chitosan and fibrin, which through Schiff‐base linkages, lent the hydrogel self‐healing properties.^67^ When seeded with vascular endothelial cells, the hydrogel allowed for the formation of capillary‐like structures. These self‐healing hydrogels have several advantages, the most obvious being that they can repair themselves following damage. In this case, the composite material was also stronger and more stable than a fibrin only material.^67^ When targeting tissue interfaces, self‐healing hydrogels can be particularly advantageous as separate hydrogel components specific to each tissue type can be placed adjacent to one another, which will then then self‐heal to form a complete construct. Indeed, Hou et al. developed an injectable self‐healing‐integrating hydrogel for regenerating the bone–cartilage interface that allowed for the relatively simple spatial localization of MSC and chondrocytes^68^ (**Figure** **6**).

Approaches that combine 3D printing with self‐healing hydrogels are also being developed. Highley et al., for example, developed a self‐healing shear thinning HA‐based bioink modified with adamantane and β‐cyclodextrin that could then be printed into a “support” HA hydrogel, creating a “guest–host” system. The printed HA‐based bioink could then be covalently cross‐linked to form a stable structure. Due to the self‐healing nature of this system, direct assembly (writing) of precise structures in 3D could be achieved that otherwise would not be possible with standard “layer by layer” 3D printing strategies.^69^

### 3.2 Integrating Biomimicry: the Importance of Mechanical and Biological Cues

In addition to mimicking the architecture of the native tissue, hydrogels used for tissue regeneration may also need to mimic other components of the tissue environment, such as mechanical and biological cues. In nature, soluble cues are transported to cells either via the vasculature or reach cells via diffusion after being secreted by their neighbors. However, cells also receive signals by interacting with their ECM. Such interactions are key in providing tissue‐specific signals to cells, instructing them to proliferate, differentiate, and secrete/degrade ECM proteins, among other cell behaviors.^70^ Indeed, within the neural stem cell niche of the subventricular zone of the brain, proteoglycans such as heparan sulfate bind many factors key for adult neurogenesis, and have been implicated in the control of proliferation and migration.^71^ Similarly, perlecan within the kidney glomerular basement membrane has been shown to play important roles in filtration.^72^

ECM‐based signals can be similarly incorporated into hydrogels, most simply by forming hydrogels from tissue‐specific ECM. This is often achieved through a two‐step process in which ECM material is solubilized into protein monomeric components, which are then formed into a hydrogel via temperature or pH‐controlled neutralization.^73^ For many complex tissues, including the heart and brain, ECM‐based hydrogels can provide important biological cues.^73^, ^74^ For example, in a stroke‐damaged rat brain model, an ECM‐derived hydrogel provided support for human neural stem cells and allowed for generation of de novo tissue.^75^ Pati et al. similarly bioprinted decellularized ECM hydrogel bioinks from heart, cartilage, and adipose tissues.^76^ When MSC or adipose‐derived stem cells were encapsulated within these decellularized ECM hydrogels, they enhanced differentiation down either the chondrogenic or adipogenic lineages, in comparison to culture within collagen hydrogels.^76^ The authors also reported enhanced myoblast maturation when cells were encapsulated in heart‐derived ECM.^76^ In short, this bioprinting strategy allowed for both the precise architectural control of the scaffold while also incorporating appropriate biological signals. Another area in which ECM hydrogels have been successful is in the formation of human breast tissue.^77^ 3D hydrogel scaffolds were formed from breast tissue‐specific ECM including both protein (collagen and fibronectin) and carbohydrate components (hyaluronan).^77^ Primary human breast epithelial cells encapsulated within the hydrogels were then shown to rapidly reorganize and form mature mammary tissue‐like structures.^77^ Importantly, tissue organization was observed in the absence of stromal cells, which are often considered key for ECM production and morphogenesis in vivo.

**Figure 7:**
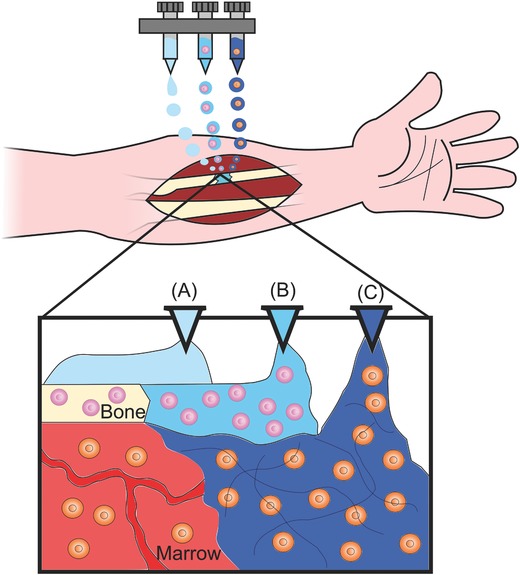
Potential strategy for in situ bioprinting. Schematic illustration showing a potential strategy for in situ bioprinting of tissue‐specific hydrogels with different cell types to treat a nonunion in the radius. Multiple hydrogels could potentially be employed to reconstruct complex, multicellular tissues like bone. A) An acellular hydrogel is printed to act as a structural support while the bone heals. B) A cell‐laden hydrogel with tissue‐specific architecture and biological factors is printed to promote ossification. C) Other cell‐laden, biologically targeted hydrogels could also be simultaneously printed to, for example, promote vascularization or aid in tissue remodeling.

One concern with ECM‐derived bioinks is that they lack structural integrity. One way this has been addressed is by printing multiple materials simultaneously: i.e., coprinting a strong synthetic hydrogel with a weaker ECM‐derived hydrogel. Indeed, with the development of multidispensing bioprinting systems, strategies such as this may be key in designing complex scaffolds with both the correct biological cues and structural integrity. One way this could be imagined is through further development of the bioprinting system developed by Kang et al. in which biological cues, such as ECM, are incorporated into the bioink, which could then be coprinted with a synthetic polymer and an outer sacrificial hydrogel mold to ensure structural rigidity (**Figure** **7**).^66^, ^76^

In addition to ECM, other biological factors can be incorporated into hydrogels to mimic biological cues of the native tissue, including growth factors and cytokines. Spatial control of these signals may be key to replicate tissue‐specific patterning during differentiation and recapitulate the heterogeneous microenvironments of the target tissue.^78^ Work by Lee and Park addressed this issue by using a thermoresponsive casting process to create precise PEG hydrogel structures with spatial distributions of different biological cues. They showed that by spatially localizing signaling molecules, MSC differentiation could be driven toward osteogenic, chondrogenic, or adipogenic lineages.^78^ Moreover, by utilizing drug‐releasing PLGA microparticles, they were also able to demonstrate sustained release of biological cues.

In addition to biological cues, mechanical cues may also be important to direct tissue formation. As discussed above, cells respond to mechanical cues in their environment, such as stiffness. Lutolf and co‐workers showed that within intestinal organoid cultures, a mechanically dynamic PEG‐based hydrogel was key in regulating appropriate cell responses. An initially stiff matrix allowed for intestinal stem cell expansion, but when switched to soft, stimulated intestinal cell differentiation.^13^ Likewise, modulation of hydrogel stiffness (7–33 kPa) has been shown to promote neocartilage formation from encapsulated cocultures of adipose‐derived stem cells and neonatal chondrocytes. In comparison to soft hydrogels, stiffer hydrogels accelerated the deposition of sulphated glycosaminoglycans, a key component of cartilage ECM.^79^

Hydrogels can also be modulated to mimic other important biological stimuli. Electrical signaling, for example, is central to normal heart tissue function and hydrogels can be designed to support this. Work by Yang et al. created a homogeneous electronically conductive hydrogel by controlling levels of conductive (Poly(thiophene‐3‐acetic acid) and flexible (methacrylated aminated gelatin) polymers. By modulating the ratio of the two components, they could precisely control the mechanical and conductive properties of the construct, achieving a Young's modulus and electrical conductivity that were both similar to that of the native heart.^80^ This advance may be crucial, as both mechanical and electrical mimicry are likely important in restoring synchronous contractile activity.^80^ Moreover, when brown adipose‐derived stem cells were seeded on the hydrogels, electrical stimulation appeared to drive cardiomyogenic differentiation.^80^ Work by Nunes et al. similarly demonstrated that electrical stimulation of 3D induced pluripotent stem cell (iPSC) cultures within a collagen gel resulted in the formation of 3D aligned cardiac tissue (“biowires”) with ultrastructural organization and electrophysiological properties that better matched those of native tissue cells compared to controls.^81^

### 3.3 Incorporating Multiple Cell Types into Complex Constructs

Besides utilizing a scaffold with appropriate mechanical and biological cues, the choice of cells in regenerative applications is essential. In addition to MSC, iPSC are also a promising option in hydrogel‐based regenerative strategies as iPSC technology allows for the development of patient‐specific cells.^82^ First described by Takahashi and Yamanaka^83^ in 2006, efforts are being made to develop robust, chemically driven protocols to differentiate iPSC into nearly every cell type in the body.^82^ As iPSC derivation and differentiation technologies have developed, so too has their use in hydrogel‐based TE strategies. For example, in a composite bioprinting strategy, a nanofibrillated cellulous alginate bioink could successfully drive chondrocyte‐derived iPSC to form cartilage‐like tissue.^84^

Cartilage TE only requires incorporating chondrocytes into a scaffold; however, for more complex tissues, multiple cell types are often necessary. Inkjet printing has been used to incorporate multiple cells types into hydrogels with precise spatial control.^85^ This was achieved in an alginate–collagen composite hydrogel system by loading different cell types (human amniotic fluid‐derived stem cells, smooth muscle cells, and endothelial cells) into separate bioink cartilages. Using a drop‐by‐drop method, cells could then be directly printed to form a complex heterogeneous construct.^85^ When implanted in a mouse model, constructs were shown to have adequate vascularization, which had previously been a hurdle for generating functional bone tissue.^85^ Another strategy to address the issues of vascularization is coprinting. This strategy has been pursued whereby different cell types, vasculature, and ECM bioinks have been coprinted to form complex heterogeneous structures^86^ and vascularized tissues as thick as 1 cm.^87^ In this technique, silicon ink is initially printed to create a customized perfusion chip. Cell‐laden inks are then printed alongside a temporary fugitive ink, encapsulated in a castable ECM. Following casting, the fugitive ink is removed leaving behind a vascular network of interconnecting channels.^87^ Tissue constructs printed in this manner demonstrated greater perfusion and survived longer culture times (6 weeks vs 14 d) than previous efforts.^86^, ^87^

### 3.4 Incorporating Cell‐Responsive and Dynamic Properties

Mechanical properties of tissues and biomaterials clearly play a role in directing cellular responses such as differentiation. However, in native tissues, many properties, including mechanical properties are dynamic. For example, during chick development, the tissue that arises from the mesoderm that is destined to become the adult heart stiffens from an elastic modulus of 0.9 to 8.2 kPa between 36 and 408 h postfertilization.^88^ Dynamic properties such as this can be incorporated into hydrogels using a number of strategies. Young and Engler, for example, matched the stiffening of their thiol‐modified HA hydrogels to that of the dynamic properties of the chick heart. They found that dynamic hydrogels upregulated the expression of mature cardiac markers when compared to cells grown on soft, but static polyacrylamide hydrogels.^88^

Other methods to incorporate dynamic properties into hydrogels include exploiting supramolecular chemistry and self‐assembly strategies. For example, by incorporating specific peptides, hydrogels can be designed to respond to physical stimuli, such as light and temperature, as well as chemical and biological stimuli, such as pH or enzyme cleavage.^52^, ^89^ Promising work by Stupp and co‐workers has shown that supramolecular nanofibers are able to effectively delivery the growth factor BMP‐2 for bone regeneration.^90^ Peptide sequences can also be used to mimic complex biological factors such as vascular endothelial growth factor (VEGF).^91^ Moreover, work by Silva et al. showed that high density presentation of a laminin‐derived epitope on supramolecular nanofibers could selectively differentiate neural progenitors into neurons.^92^ Indeed, incorporation of such supramolecular materials into hydrogels for controlled delivery may enable the development of dynamic systems in which the materials themselves are capable of adapting to their surrounding environment.

In addition to supramolecular materials, researchers are also exploring dynamic materials based on 4D biomimetic printing. For example, researchers have used cellulose fibrils to print anisotropic swelling behavior into hydrogels that change their shape as a function of time following immersion in water.^93^ The authors used this system to create nature‐inspired 3D shapes, such as that of the *Dendrobium helix*, a stunning orchid native to the lowlands of New Guinea. However, such technologies could also potentially be applied to bioprint tissues with mesoscale structures that form upon immersion in biological fluids.^93^ While 4D biomimetic systems for regenerative applications have not yet been reported, we envision a future that would utilize such technologies for clinical applications. For example, there has been much effort devoted to creating TE heart valves to replace stenotic or damaged values resulting from disease or congenital conditions. However, such advanced therapies would likely still require traumatic and dangerous open‐heart procedures to surgically implant them. A 4D bioprinted heart valve, on the other hand, may allow for the future development of minimally invasive heart valve repair whereby a new valve could be inserted via a catheter and formed in situ in the heart. Similarly, maxillofacial surgeries to repair orbital floor fractures, common in patients in motorbike accidents, are often necessary to prevent enophthalmos, but can suffer from complications sometimes associated with the surgical placement of alloplastic implants.^94^ Here, a future in which 4D‐bioprinted constructs, fabricated to match a patient's own facial structures based on high‐resolution imaging, and placed in a minimally invasive procedure prior to assuming their full shape, are feasible.

## 4 Acellular Hydrogel Approaches

**Figure 8:**
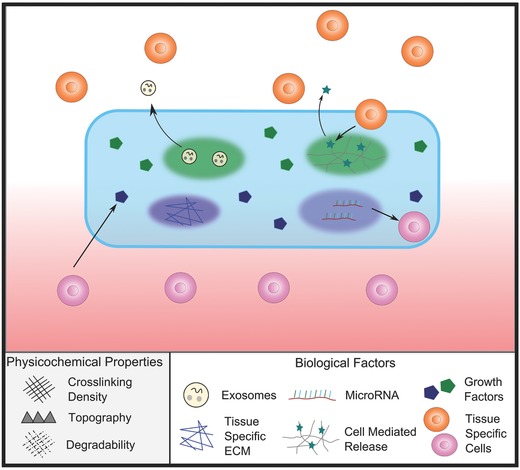
Using acellular hydrogels to promote tissue regeneration. For regenerative strategies based on acellular hydrogels, various factors can be incorporated into the hydrogel. Such factors may include growth factors, regenerative exosomes, or microRNA, which will be released over time as the hydrogel degrades. Acellular hydrogels can also be used to directly instruct host cells. For example, tissue‐specific ECM or topography can also be incorporated into the hydrogel to direct host stem cell differentiation as cells come in contact with it. More advanced acellular hydrogel strategies might incorporate controlled spatial and temporal release of specific factors. Using cleavable systems such as those mediated by MMP activity, factors will only be released in response to cell‐ or tissue‐specific stimuli. This could be particularly useful for tissue interfaces such as the Achilles tendon‐bone insertion (enthesis), where the spatial distribution of biological factors is key in modulating cell behavior.

As cell‐based therapies such as TE offer numerous possibilities for tissue regeneration, there is good reason for excitement surrounding their development. However, cell‐based therapies face significant challenges in terms of cost, regulatory hurdles, and scalability.^95^, ^96^ Cell free therapies, on the other hand, are often not as limited in their path to clinical translation because they tend to be less complex. Traditionally, acellular biomaterials have been used simply as fillers and for structural support, however, a new generation of acellular hydrogels are being designed to interact with endogenous factors, including local tissue and cells, to aid with healing and promote tissue regeneration (**Figure** **8**).^95^, ^97^

### 4.1 Hydrogels as Controlled Delivery Vehicles for Bioactive Molecules

The fundamental approach of many acellular biomaterial strategies is to trigger tissue regeneration in situ by utilizing the body's own regenerative capabilities. This is often achieved by mobilizing host tissue progenitors to the site of injury or prompting nearby cells to mediate repair. The concept of biomaterial‐driven tissue regeneration is not new and many biomaterials have been designed to deliver biological molecules such as growth factors and cytokines to stimulate matrix production and cell growth. There are many excellent reviews available on this subject; therefore, we will only comment on a limited number of strategies here.^98^

One of the most well‐known drug delivery platforms based on a biomaterial is Medtronic's INFUSE system, which comprises a collagen type I biomaterial that releases recombinant human BMP2, and is placed surgically to treat long bone fractures and in spinal fusion procedures.^99^ Although the INFUSE system has been highly criticized for causing side effects such as inflammation when used in off‐label procedures,^100^ the fundamental idea of using a biomaterial to deliver an active biological molecule is widespread. Indeed, many issues associated with the off‐label use of INFUSE arose from the high dosage of growth factors rather than delivery strategy itself.^100^ Cardiac stents have been similarly designed to elute drugs that can prevent re‐stenosis of coronary arteries,^101^ and wound dressings which release antibiotics and growth factors to aid healing are widely described.^102^

Hydrogels provide an attractive system for the delivery of biological molecules, as in addition to good biocompatibility and amenability for minimally invasive delivery, they are also highly physically and chemically modifiable,^98^ which can allow for precise control of release of biological molecules over minutes, hours, days, and perhaps even years. Most commonly, this release rate is mediated via diffusion, which can be easily controlled by varying either the hydrogel's network size or degradation rate.^98^ One strategy to control hydrogel degradation involves a similar strategy to that used to create degradable ex vivo tissue models: cross‐linking hydrogels with peptides that are susceptible to cleavage by MMPs.^103^ For example, MMPs are important in heart tissue homeostasis, but in specific cardiovascular diseases excessive MMP production can lead to inflammation and tissue damage. Purcell et al. exploited this tissue response with a polysaccharide‐based hydrogel that electrostatically sequestered a recombinant MMP inhibitor. The hydrogel was then cross‐linked with MMP‐cleavable peptides such that cleavage of the peptides mediated the release of the MMP inhibitor.^104^ When placed in a porcine model of myocardial infarction, the hydrogel was effective in reducing adverse left ventricular remodeling.^104^

As our understanding of the biological mechanisms of tissue regeneration continue to grow, so too has the development of hydrogels that aim to exploit them. One way this has been pioneered is through understanding the paracrine mechanisms by which stem cells can stimulate tissue regeneration. For example, MSC appears to mediate regeneration via release of paracrine factors rather than de novo regeneration.^105^ Hydrogels provide an exciting opportunity in this context as they can allow for the controlled delivery of cell‐derived regenerative factors, such as exosomes, without the regulatory hurdles of delivering cells themselves. This was demonstrated recently by Tao et al. who employed a chitosan hydrogel that mediated sustained release of MSC exosomes. When tested in a rat model, the material accelerated skin wound healing.^106^

### 4.2 Exploiting Hydrogel Physical Properties to Direct Host Cell Response

In addition to releasing bioactive factors that can regulate regeneration, researchers are also developing hydrogels with physical properties, such as stiffness, degradability, and topography, that are matched to those of the native tissue or can recruit and/or direct cells in regeneration.^107^, ^108^ One area in which this has been particularly successful is in CNS regeneration, as hydrogels can be designed to have similar mechanical properties to those of the brain.^108^ HA‐based hydrogels with elastic moduli in the range of 3–10 kPa have been shown to be optimal for neural progenitor cell (NPC) differentiation.^109^ NPC encapsulated in soft hydrogels that mimicked the stiffness of a neonatal brain (≈2.6 kPa) differentiated into neurons. However, when encapsulated in slightly stiffer hydrogels, more akin to that of adult brain (≈5.7 kPa), NPC differentiated into astrocytes.^109^ For CNS regeneration, hydrogel degradation is also key. Silk‐based hydrogels have been shown to be advantageous in this context as they have slow degradation rates that may foster neural regeneration.^110^

Other physical features of hydrogels can also be exploited to direct host cells. In addition to degradability, cell infiltration into an acellular hydrogel will also depend on hydrogel porosity/mesh size, the availability of cell adhesive motifs—often adhesive peptides, syndecans,^111^ or cadherins^112^—and the hydrogel's amenability to promote the formation of a vasculature. Cells need to be within ≈100 to 200 µm of a blood vessel in order to receive enough nutrients and oxygen for normal function.^113^ Hydrogel architecture appears to play an important role in modulating this. Chiu et al.^114^ showed that pore sizes within PEG hydrogels between 50 and 150 µm in diameter allowed for the formation of vasculature, while pore sizes of 25–50 µm limited vessel infiltration.^114^ Micropatterning techniques have also been exploited to guide cell migration in acellular hydrogels. Lee et al. demonstrated a photo laser scanning photolithography technique in which RGD sites could be patterned into a collagenase‐sensitive poly(ethylene glycol‐co‐peptide) diacrylate hydrogel to direct cell migration.^115^ They showed that cell migration along patterns promoted successful wound healing.

### 4.3 Hydrogels with Immunomodulatory and Gene Therapy Functionality

The immune system plays an important role in tissue repair and is emerging as a key target of biomaterial‐based regenerative strategies. Some biomaterials are known to interact directly with particular aspects of the immune system.^116^ HA, for example, has anti‐inflammatory properties, while natural materials such as chitosan scavenge free radicals, which can then reduce/suppress inflammation.^116^, ^117^ Indeed, active control of the immune system may be key in modulating tissue repair and regeneration. Inflammation is an essential response to injury, and plays a central role in initiating healing.^116^ During normal healing, acute inflammation is followed by a resolving anti‐inflammatory response, and the restoration of tissue integrity.^116^ Issues arise if inflammation becomes chronic and inhibits healing.^118^ This scenario provides an opportunity for hydrogels with dual functionalities that can help mediate these responses. For example, an initial hydrogel‐based delivery of a proinflammatory signals such as SDF‐1 (an inflammatory and angiogenic cytokine) might aid in the mobilization of progenitor cells.^119^ This response could then be followed by the release of anti‐inflammatory mediators such as IL‐4 and IL‐10, which are key for tissue repair as they trigger macrophages to switch from a proinflammatory to a reparative phenotype.^120^ Similarly to the MMP‐cleavable systems that have been exploited to treat the effects of myocardial infarction,^104^ such systems could be designed to release a proinflammatory molecule until an appropriate response from the native tissue triggers release of an antiinflammatory molecule to resolve tissue healing.

In addition to releasing inflammatory molecules, hydrogels can also be used to modulate the host's immune system. For example, biomaterials can be used induce an antigen‐specific tolerogenic response to treat a variety of autoimmune diseases.^121^, ^122^ This was demonstrated by Verbeke et al. who used an alginate hydrogel to deliver a peptide antigen mimotope to treat type 1 diabetes in a mouse model. This peptide hydrogel delivery system resulted in expansion of antigen specific T cells in the lymph nodes.^122^ Beyond treating autoimmune diseases, similar therapies could also be used to modulate transplant rejection.^122^

Gene therapy technologies similarly hold great promise for regeneration.^123^ With such therapies, successful delivery is vital, making hydrogels an attractive platform to locally and sustainably deliver appropriate molecules. For example, Yang et al. used a PECE thermoresponsive hydrogel to deliver an antioncogene. This system was capable of sustainably and locally delivering the gene, which was important for maximizing its antitumour effects, but minimizing systemic side effects.^124^ Hydrogels have also proven useful as lentiviral delivery systems, and there is evidence that they both increase the stability of the virus as well as help protect it from the immune system.^125^ Indeed, for lentiviral gene therapy, host immune response can affect efficacy. Hydrogels with small pore sizes can limit complement and antibody diffusion, potentially protecting the virus.^125^ Hydrogels have been used for the delivery of nucleic acids to treat spinal cord injury.^126^ Aligned nanofiber hydrogel scaffolds were shown to sustainably release microRNAs that enhanced axon regeneration.^126^ Scaffold design here mediated alignment of the fibers by providing topographical cues to direct neurite extension.^126^ With the development of CRISPR/Cas9 systems, hydrogels may also have the potential to play an important role in genome editing technologies in the future.

## 5 Hydrogel Delivery Strategies

**Figure 9:**
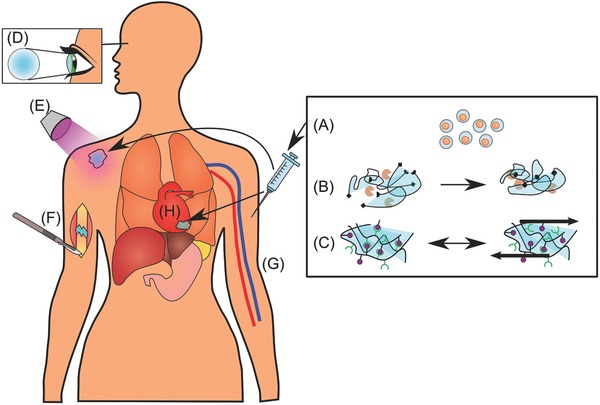
Various strategies for hydrogel delivery. Schematic diagram showing a range of strategies for hydrogel delivery and gelation. A) Single cells can be encapsulated in thin hydrogels, which can then be injected intravenously to be distributed throughout the body. The hydrogel coating can be designed to protect the cells from the immune system. B) Hydrogels can be covalently cross‐linked by enzyme‐mediated reactions between reactive groups on the hydrogel monomers, forming a network. C) Noncovalent cross‐linking of hydrogels can be achieved via hydrophobic, interactions, π–π interactions, hydrogen bonding, metal chelation, or van der Waals interactions. These hydrogels can sometimes be shear thinning, which makes them easily injectable and may also protect encapsulated cells during the injection process. D) Hydrogels can be used as cornel implants and keratoprostheses. E) UV light can be used to cure hydrogels subcutaneously after injection or activate biological moieties like cell‐adhesion peptides, providing spatiotemporal control of their biological activity. F) Surgical implantation of a hydrogel to treat a nonunion fracture. G) Micro‐ and nanohydrogels, with or without cells, can be delivered via IV injection. H) Hydrogel patches with encapsulated cardiac stem cells can be surgically implanted to treat myocardial infarction.

While researchers exploit hydrogel technologies to understand how biochemical and biophysical cues influence cell and tissue function to create 3D tissue models and TE platforms, being able to exploit these cues with hydrogels depends upon our ability to deliver them for appropriate therapeutic applications (**Figure** **9**). Simple implantation of biomaterials through traditional surgical means is a standard delivery route. However, surgery can lead to morbidity at the implantation site, is associated with surgical and recovery costs, and will almost always cause patient discomfort.^127^ For example, to treat osteochondral defects on the articulating surfaces of the knee, early hydrogel implants (such as Cartipatch, an alginate‐based construct) required surgery, which could cause inflammation and increased the chance of infection, which could result in treatment failure.^128^ To overcome these drawbacks there have been significant efforts to design injectable, in situ forming, and nano/microhydrogel systems that can be delivered in a minimally invasive manner.

For any hydrogel‐based therapeutic to be a viable clinical option, the cross‐linking/gelation mechanism must be biologically mild as to not damage the surrounding tissue or biologics, including cells, in the hydrogel. Injectable hydrogels that have been developed for tissue repair employ various gelation methods that are mediated through chemical or physical interactions. Deciding which gelation mechanism to use depends on the application and the desired effect. Chemical interactions are covalent bonds formed between precursors that cross‐link the hydrogel. Covalently formed hydrogels are often robust and mechanically strong. For example, Michael addition reactions between thiol groups and acrylates or methacrylates have been employed to form covalently cross‐linked hydrogels and are often aided by UV light.^129^ Physical interactions, on the other hand, lead to hydrogels that have reversible bonds between hydrogel components, and are achieved using molecular interactions like hydrophobic interactions, π–π interactions, hydrogen bonding, metal chelation, and van der Waals interactions.^52^ Covalent cross‐linking typically requires physical initiators while noncovalent cross‐linking often arises spontaneously under specific conditions.^108^ A combination of both covalent and physical interactions in the same hydrogel has also been explored^130^, ^131^ to create hydrogels for cartilage^132^ and cornea repair.^133^ For example, a network that contains both rigid and ductile components can be mechanically advantageous because the rigid network can sustain load, while the ductile network dissipates energy, preventing failure.^130^ Indeed, Shin et al.^130^ created a hydrogel that was made of a covalently linked gellan gum network, which had a second covalently linked gelatine network incorporated within it. This biocompatible, double network could be loaded with cells, and showed excellent load‐bearing properties, approaching those of native articular cartilage.

### 5.1 Injectable Hydrogels Cross‐Linked by Light

UV irradiation‐induced cross‐linking is a common approach to covalently cross‐link hydrogels or activate biologics in hydrogels in situ.^134^, ^135^, ^136^ UV cross‐linking allows quick gelation within seconds^137^ to minutes.^138^ This characteristic is important as it provides a means to quickly solidify a liquid precursor, which otherwise may be diluted by blood or other body fluids. As well as possessing quick gelation times, UV‐cross‐linkable hydrogels can be designed with low molecular weight, and thus low‐viscosity, precursors. This is advantageous because it allows the hydrogel to be injected and then take the shape of a defect or cavity prior to cross‐linking. This space filling property may be crucial for proper integration with surrounding tissues and regeneration.

Many types of biological and synthetic molecules/polymers can be delivered and cross‐linked via light, including polyethylene glycol,^139^ poly(2‐hydroxyethyl methacrylate),^140^ modified HA,^141^ and modified chitosan.^142^ The ability to covalently incorporate various moieties to mediate cross‐linking and/or incorporate biological components is commonly achieved by attaching acrylate, vinyl, methacrylate, or thiol groups to these molecules or taking advantage of similar side groups. UV irradiation‐inducible cross‐linking is often facilitated by non‐toxic photo initiators like Irgacure 2959,^143^ which is part of the free radical‐dependent Michael addition that polymerizes the hydrogel via the incorporated acrylate, vinyl, methacrylate, or thiol groups. UV irradiation can also be used to spatially control specific features like stiffness^144^ and the presentation of bioactive molecules.^145^

The versatility and adaptability of light‐mediated cross‐linking make it particularly attractive when developing hydrogel‐based therapies for in vivo use. Lin et al.^146^ engineered an injectable, cell‐laden hydrogel based on methacrylated gelatine. After subcutaneous injection, the hydrogel was rapidly cross‐linked via UV irradiation and Irgacure 2959 through the skin. After 7 days, the hydrogels supported the formation of a stable vasculature if endothelial colony‐forming cells or MSC were incorporated. UV‐curing hydrogels have also been explored to cross‐link an injectable hydrogel transdermally to deliver model proteins in a mouse model.^134^

While UV irradiation can be used to cross‐link hydrogels in situ, Lee et al. have shown how a similar technique can be used to activate bioactive components in an implantable hydrogel.^147^ This was achieved with a PEG‐based hydrogel modified with RGD sequence‐containing peptides that were protected by a 3‐(4,5‐dimethoxy‐2‐nitrophenyl)‐2‐butyl ester photolabile caging group, which could be released upon exposure to UV light. Using this chemistry, they spatially and temporally activated the RGD peptides using precise and timed exposure to UV light that released the molecular cage. In a transdermal mouse model, the application of UV light was able to regulate cell adhesion, inflammation, fibrous encapsulation, and vascularization.^147^ This spatiotemporal control may have important implications for researchers' ability to direct tissue repair because it has the potential to allow for the controlled formation of vasculature and tissue heterogeneity.

Nevertheless, while UV‐mediated cross‐linking has numerous advantages, including that it allows for fast, spatial, and temporal control of gelation, UV light curing methods may impact cell viability and negatively affect surrounding host tissue due to the formation of radicals, and to a lesser extent, from the UV light itself.^134^, ^143^, ^148^ This method is also limited by the depth UV irradiation can travel through tissue and the amount of UV exposure and radicals a tissue can withstand without damage. Indeed, UV‐induced gelation is unlikely to see applications deep in tissues. To overcome potential toxicity associated with UV light curing methods, systems which employ visible light to initiate cross‐linking have also been developed.^149^ For example, Fu et al. developed a heparin/PEG‐based visible light (525 nm) cross‐linkable hydrogel that uses eosin Y and triethanolamine as photoinitiators. In this system, thiolated‐heparin was cross‐linked with PEG diacrylate through a Michael‐type addition to form a covalently cross‐linked network. Encapsulated fibroblasts remained viable in these hydrogels and growth factor loading was comparable to that achieved in other heparin‐based systems.^150^

### 5.2 Stimulus‐Driven In Situ Forming Hydrogels

Another approach to deliver therapeutic hydrogels is by harnessing their potential for in situ cross‐linking. Unlike UV irradiation‐induced cross‐linking systems, which use external stimuli, in situ forming hydrogels rely on specific physiological conditions (pH/temperature),^151^ delayed/secondary cross‐linking mechanisms,^136^ or enzyme‐mediated cross‐linking^152^ to trigger hydrogel formation. This type of delivery approach has many of the benefits of UV cross‐linked systems, like injectability, and fast gelation, but often lacks amenability for spatiotemporal control of gelation. Some key advantages of the in situ forming hydrogels are their ease of use in surgery as well as their adaptability to various applications, such as in deep tissues where light‐mediated methods are not feasible.

Enzymatically cross‐linked hydrogels are one promising form of in situ cross‐linked hydrogels. These hydrogels are polymerized through the catalytic activity of enzymes added exogenously, which facilitate the covalent bonding between two substrates that are linked to larger molecules that comprise the bulk hydrogel. Enzymes that have been explored to induce gelation include transglutaminase, tyrosinase, phosphopantetheinyl transferase, lysyl oxidase, plasma amine oxidase, phosphatases, thermolysin, β‐lactamase, phosphatase/kinase, and peroxidases.^153^ Enzymes of particular interest are transglutaminases, which cross‐link via the formation of ε‐(γ‐glutamyl)lysine bonds or the incorporation of primary amines with glutamine residues.^154^ Transglutaminases are advantageous because they can facilitate a tight integration of the hydrogel with the surrounding host tissue. This tight integration between the native tissue and hydrogel is due to the readily available naturally occurring substrate for transglutaminases, which exists in native tissues.^155^

A novel use of enzyme‐mediated cross‐linked hydrogels was recently reported by Griffin et al.,^156^ who engineered PEG‐based microhydrogel beads that were modified by covalently attaching glutamine and lysine. These molecules can act as substrates for factor XIII, a transglutaminase enzyme involved in blood coagulation. Upon addition of exogenous factor XIII, the hydrogel beads were cross‐linked via reactions between the primary amine on the lysine with the glutamine to produce porous hydrogels that human dermal fibroblasts, adipose‐derived MSC, and bone marrow‐derived MSC could infiltrate. The authors also injected these materials into a mouse skin wound model and cross‐linked them with exogenous factor XIII in situ to form a porous scaffold. Wounds treated with the cross‐linked microhydrogels showed faster wound closure and decreased inflammation compared to those treated with a nonporous hydrogel made of the same material. Other groups have also used enzyme cross‐linked hydrogels for cartilage TE. For example, gelatin‐hydroxyphenylpropionic acid has been cross‐linked with horseradish peroxidase catalyzed by H_2_O_2_ to form a viscoelastic hydrogel. When these enzyme cross‐linked hydrogels were used to encapsulate chondrocytes and then injected into an osteochondral defect in a rabbit, improved cartilage regeneration was reported in comparison to that in no hydrogel controls.^157^

Nevertheless, while enzymatically cross‐linked hydrogels show promise, the inclusion of exogenous enzymes may have unforeseen deleterious biological effects. For example, exogenous application of transglutaminases in vivo most likely also cross‐links ECM proteins in the tissue surrounding the hydrogel, which can lead to tissue stiffening. Indeed, while transglutaminases are essential for many biological processes, they can also contribute to the pathophysiology of various inflammatory, autoimmune, and degenerative conditions.^158^

### 5.3 Nonstimulus‐Driven In Situ Cross‐Linking Hydrogels

Hydrogels covalently cross‐linked in situ via nonstimulus driven mechanisms provide another option to deliver therapeutic hydrogels. These hydrogels are typically composed of precursor components that are mixed immediately before or during injection, often rely on simple gelation mechanisms, and are easy to use. One in situ cross‐linking method that has shown promise is click chemistry. Click chemistry refers to a number of different types of chemical reactions that are highly efficient, even at low concentration. This is a particular advantage for biological hydrogels because of the low solid content of most systems. Click reactions are also advantageous because of their quick reaction rates and biocompatible reaction conditions. Unlike other mechanisms, such as enzyme‐mediated cross‐linking, click reactions are highly selective chemically, meaning that they do not readily produce unwanted side reactions. They have also been shown to be compatible with cells, drugs, and proteins.^159^ Hermann et al.,^160^ for example, developed an injectable click‐based hydrogel to delay bone growth in a mouse calvarial model in which rapid regrowth of bone can lead to craniofacial deformities, restricted brain growth, and an increase in intracranial pressure. They utilized multivalent PEG precursors that formed a network upon mixing via ring‐strain promoted Cu‐free reactions between dibenzylcyclooctynes and azides attached to the PEG. Importantly, by incorporating the BMP antagonist Gremlin into their hydrogel, they were able to delay bone re‐growth, demonstrating a potential therapy to control bone over‐growth and reduce the risk of life threatening complications.^160^

As well as being able to form hydrogels to deliver biologics, click chemistry has also been exploited to construct microporous hydrogels that when formed in the presence of cells, create complex polymer–cell composite systems with variable pore sizes.^161^ To accomplish this, microgels were formed via an inverse suspension polymerization method with either dibenzocyclooctyne or azide groups. When these two microgels were combined, they formed a covalently connected microgel network that could entrap cells. This microgel system provided tunable properties that controlled cell–material interactions and cell morphology.

### 5.4 Intravenous Delivery of Cellular Nano‐/Microgels

Although there have been a plethora of clinical trials with MSC, it is clear that simply injecting cells intravenously limits their therapeutic value.^162^ Injected cells often can only briefly participate as immunomodulatory or signaling cells before they are cleared from the body.^163^ While many of the hydrogels discussed so far have focused on bulk materials that provide physical support to cells, techniques have also been developed to encapsulate cells within a thin layer of hydrogel to facilitate cell‐based therapies.^164^ The goal of this approach is often to deliver signaling cells that can modulate immune or regenerative responses via paracrine signaling, or signal to other therapeutically relevant cells/recruit them to a specific site to participate in repair. For example, single MSC have been encapsulated with a 5.8 µm thick sodium ion cross‐linked alginate hydrogel coatings to form cell‐containing microgel beads. When delivered intravenously in a mouse model, encapsulated cells remained in the mouse for longer and were shown to provide a longer sustained release of cell‐secreted factors than cells injected without coating.^163^

One drawback of intravenous injection is that radially injected cells are often trapped in the lungs,^165^ likely due to their size.^166^ Cells coated with a thin hydrogel, however, may fair a better chance of evading immune system clearance as hydrogels can be designed to preclude cell detection or simply provide a physical barrier that discourages engulfment. Hydrogels may also provide other interesting solutions to the problem of pulmonary passage. By incorporating small molecules or biologics on the surface of cell containing microgels, hydrogels could passively direct encapsulated cells to an area of damage or disease before they reach the lungs. Hydrogels could also act as homing vehicles to direct therapeutically relevant cells to specific locations in the body.

## 6 Clinical Translation of Hydrogel‐Based Therapies

The majority of the drugs and therapeutics developed for human use fail during their discovery and development stages. Indeed, only 10.4% of all candidates put through Phase I clinical trials eventually receive approval for use in humans.^167^ For more than 20 years now, TE‐based therapies have been proposed and pushed through the development stages toward clinical translation in the hope of creating functional tissues to replace those lost to disease or injury.^168^ As of July 2017, there were 371 clinical trials registered worldwide that related to hydrogels and 69 that focused on TE (www.clinicaltrials.gov). Although only a handful of these studies aimed to apply recent advances in hydrogels to TE, there have been some limited successes, particularly for planar and hollow organs, such as skin, cornea, urethra, urinary bladder, and blood vessels.^169^ However, the development of TE‐based regenerative strategies for more complex tissues still faces a number of key challenges.^168^, ^170^ These include: (1) provision of adequate oxygen and nutrients to large tissues, which likely require the formation of a complex vasculature; (2) incorporation of multiple cell types with precise spatial arrangements; (3) achievement of appropriate, tissue‐specific mechanical properties (such as stiffness, shear strength and hardness); and (4) integration of TE constructs with surrounding tissue. Hydrogel‐based TE scaffolds offer the possibility of addressing many of these challenges and perhaps can be successfully translated into viable therapies. We highlight some promising preclinical and clinical studies that have exploited hydrogels for TE‐based therapies below.

### 6.1 Cartilage Repair

Articular cartilage is an obvious target for clinical repair using hydrogel technologies. The market for cartilage repair is tremendous given that 13.8% of the adult population over the age of 60 suffers from osteoarthritis, a number that will increase as the global population ages.^171^ Articular cartilage is amenable to TE strategies because it lacks some of the challenges of more complex tissues because it is, for the most part, avascular, aneural, and only contains a single type of cell. Much research has been undertaken with hydrogels to regenerate cartilage,^172^ including using naturally derived ECM‐based hydrogels such as type I collagen, and thermoresponsive hydrogels formed from chitosan/PVA composites. There are also reports of efforts to engineer cartilage with synthetic hydrogels,^173^ including one based on a PLGA–PEG–PLGA hydrogel.^174^

One particularly promising clinical application of a synthetic hydrogel for cartilage repair was reported by Sharma et al. in 2013.^4^ They developed a photoreactive, adhesive, PEGDA‐based hydrogel, which they injected into focal cartilage defects in the medial femoral condyle of 15 patients in conjunction with standard microfracture surgery. Although the injected hydrogel itself was acellular, the concomitant microfracture surgery allowed autologous cells to invade into the hydrogel. When compared to outcomes in patients treated with microfracture alone, those who received the hydrogel‐based therapy showed greater tissue fill and increased tissue organization (both by MRI). Patients also reported less pain, an important clinical outcome in this patient group. Although long‐term follow‐up is required to determine if the benefits of the treatment persist, and importantly, if the treatment ultimately prevents patients with focal lesions from going on to develop osteoarthritis, results appear promising.

A number of preclinical trials in cartilage regeneration using hydrogels have also been reported. For example, researchers incorporated kartogenin and MSC into a synthetic PLGA–PEG–PLGA hydrogel and showed good cartilage repair in a rabbit model.^174^ Similarly, a hydrogel formed from oligo[poly(ethylene glycol)] fumarate combined with encapsulated MSC, was reported to mediate a more hyaline‐like repair than implantation of the scaffold alone in a porcine model.^175^

### 6.2 Cardiovascular Regeneration

The other application of hydrogels for TE that dominates preclinical and clinical trials is for cardiovascular applications. Cardiovascular diseases place a tremendous burden on society and are the leading cause of mortality worldwide.^176^ Particularly in the United States and Europe, around 5% of the acute hospital admissions are due to cardiac events, and 10% of hospitalized patients suffer from some form of cardiovascular disease.^176^

In a completed Phase I study (NCT00981006), a gelatin hydrogel sheet that allowed for the controlled release of bFGF (200 µg) was combined with autologous cardiac‐derived stem cells (5 × 10^5^ cell kg^−1^) to treat six patients with ischemic cardiomyopathy following acute myocardial infarction.^177^ In this study, Takehara et al. showed that the therapy was safe and feasible. However, while some benefits were noted 6 months posttreatment, the sample size was too small to conclude efficacy.^177^ Similarly, a phase I clinical trial (NCT02057900) led by Assistance Publique–Hôpitaux de Paris investigated the feasibility of human embryonic stem cell (ESC)‐derived CD15+ Isl‐1+ progenitors embedded within a fibrin hydrogel patch to treat patients with ischemic heart failure.^178^ In this study, a fibrin hydrogel patch was used, as it had been shown previously in animal models to play an essential role in improving cell retention and survival.^179^ While no conclusions could be drawn on efficacy, overall patient functional outcomes were promising.^178^

Despite some promising results, the overall efficacy of cardiovascular cell therapies is inconclusive. A meta‐analysis that examined treatments for heart failure showed that among 31 randomized cell therapy trials comprising 1521 patients, exercise capacity, left ventricular ejection fraction, and quality of life were all improved in the treated patients.^180^ However, a second meta‐analysis that examined patient data from 12 trials could find no benefit of intracoronary cell therapies in treating acute myocardial infarction.^181^ Such conflicting data raise concerns about cell‐based therapies for cardiovascular diseases and highlight the need for improved strategies. For future treatments, multidisciplinary approaches may provide an answer. Cell retention in cardiac tissue, for example, is thought to be important, and hydrogels have the capability to improve this.^182^ Successful differentiation into mature cardiomyocytes is likely also key, and again, could be aided by careful scaffold design. Indeed, many cell types are known to be mechanoresponsive, including cardiomyocytes. Work by Morez et al. showed that modifying material surface topography by creating microfabricated grooves could promote cardiac progenitor elongation and alignment, driving appropriate differentiation.^183^ Cell‐containing cardiac patches that rely on bioprinting technologies are similarly promising. Using a bioprinting strategy, precise patterns of human umbilical vein endothelial cells and hMSC were created; and when implanted in a rat model of myocardial infarction, led to increased angiogenesis and improved cardiac function in comparison to patches in which cells were seeded randomly.^184^ Another consideration for successful treatment of myocardial infarction is delivery time. A disadvantage of patch‐based delivery systems is that they often require surgical procedures. Injectable hydrogels, on the other hand, can be delivered quickly to the site of injury.^185^ Indeed, injectable hydrogels could be delivered minimally invasively as transcoronary infusions or transendocardial injections.^185^ Such fast delivery of cells/scaffolds may help prevent damage to the heart tissue postinfarction. While no such injectable hydrogel systems have been used clinically, future developments in dynamic, injectable hydrogels may make this achievable.

## 7 Conclusions

Over the last decade, hydrogel technologies have improved dramatically allowing researchers to create ex vivo tissue models that replicate that native tissue better than ever before. Researchers are also developing hydrogel‐based biomaterials with controlled architectures and biological and physical properties that can be used for TE. Moreover, acellular hydrogels that can deliver bioactive molecules are increasingly finding use in drug delivery applications, often relying on their injectability and chemistries that allow for in situ gelation. Taken together, the result is a toolbox of hydrogel‐based materials that can be used by both bench‐based researchers to answer fundamental questions in cell biology, and physicians to either replace damaged tissues or deliver cells/molecules to mediate repair. Indeed, the advancements in regenerative medicine that these new hydrogel technologies are likely to foster are surely something to feel “swell” about.

Despite such exciting developments, however, developments in hydrogel research only seem to trickle into a few limited clinical applications. This may be partly attributable to regulatory and funding limitations, but may also be because of risk. Tissue regeneration is clearly a complex process, which will require complex biological and material‐based strategies to tackle. Indeed, it is likely that only through careful consideration of the both the target tissue's biological, physical and mechanical properties can hydrogels be developed that can truly mediate or participate in regeneration. However, as fundamental research in cell biology reveals how cells respond to their ECM and their niche, and new insights into endogenous tissue regenerative mechanisms are identified, materials scientists and chemists will inevitably develop hydrogels that can exploit and target them for regeneration.

## Conflict of Interest

The authors declare no conflict of interest.
